# (–)-Epicatechin Reduces the Blood Pressure of Young Borderline Hypertensive Rats During the Post-Treatment Period

**DOI:** 10.3390/antiox9020096

**Published:** 2020-01-23

**Authors:** Michal Kluknavsky, Peter Balis, Martin Skratek, Jan Manka, Iveta Bernatova

**Affiliations:** 1 Slovak Academy of Sciences, Centre of Experimental Medicine, Institute of Normal and Pathological Physiology, 813 71 Bratislava, Slovakia; michal.kluknavsky@savba.sk (M.K.); peter.balis@savba.sk (P.B.); 2 Slovak Academy of Sciences, Institute of Measurement Science, 841 04 Bratislava, Slovakia; martin.skratek@savba.sk (M.S.); jan.manka@savba.sk (J.M.)

**Keywords:** (–)-epicatechin, borderline hypertensive rats, nitric oxide, redox balance, iron, *Nrf2*, *PPAR-γ*, open field

## Abstract

This study investigated the effects of (–)-epicatechin (Epi) in young male borderline hypertensive rats (BHR) during two weeks of treatment (Epi group, 100 mg/kg/day p.o.) and two weeks post treatment (PE group). Epi reduced blood pressure (BP), which persisted for two weeks post treatment. This was associated with delayed reduction of anxiety-like behaviour. Epi significantly increased nitric oxide synthase (NOS) activities in the aorta and left heart ventricle (LHV) vs. the age-matched controls without affecting the brainstem and frontal neocortex. Furthermore, Epi significantly reduced the superoxide production in the aorta and relative content of iron-containing compounds in blood. Two weeks post treatment, the NOS activities and superoxide productions in the heart and aorta did not differ from the age-matched controls. The gene expressions of the NOSs (*nNOS*, *iNOS*, *eNOS*), nuclear factor erythroid 2-related factor 2 (*Nrf2*), and peroxisome proliferator-activated receptor-γ (*PPAR*-γ) remained unaltered in the aorta and LHV of the Epi and PE groups. In conclusion, while Epi-induced a decrease of the rats’ BP persisted for two weeks post treatment, continuous Epi treatments seem to be necessary for maintaining elevated NO production as well as redox balance in the heart and aorta without changes in the *NOSs*, *Nrf2*, and *PPAR*-γ gene expressions.

## 1. Introduction

Borderline hypertension, i.e., prehypertension, was first defined in the seventh report of the Joint National Committee on Prevention, Detection, Evaluation, and Treatment of High Blood Pressure, as being characterized by systolic blood pressure (BP) between 120 and 139 mm Hg or diastolic BP between 80 and 89 mm Hg [1]. Data from the 1999–2008 that were published by the National Heart, Lung, and Blood Institute showed the prevalence of prehypertension in the young US population (18–29 years old) ~ 26% and ~33% among those aged 40–49 years [2]. High prevalence of prehypertension has been found in school-aged and adolescent populations [3]. In addition, several studies have confirmed the elevated risk of cardiovascular diseases in prehypertensive people [4,5]. These findings have led to the formulation of new guidelines for the prevention, evaluation, and management of people with systolic BP above 120–139 mm Hg. 

There are several terms for systolic BP that are in the range of 120–139 mm Hg. The European Society of Cardiology and European Society of Hypertension defined systolic BP of 120–129 mm Hg as “normal” and 130–139 mm Hg as “high normal” [6]. The American College of Cardiology and American Heart Association defined systolic BP of 120–129 mm Hg as “elevated” and 130–139 mm Hg as “stage 1 hypertension” [7]. Both of the guidelines currently recommend non-pharmacological interventions for each stage of elevated systolic BP and pharmacological treatment for high normal/stage 1 hypertension (i.e., systolic BP in the range of 130–139 mm Hg) if other risk factors are present, despite the differing terminologies [6,7]. There is a considerably lower number of experimental studies focused on the mechanism of preventing high BP during the prehypertensive phase than studies focused on the treatment of fully developed hypertension, despite the high prevalence of prehypertension among humans.

One of the experimental models of essential prehypertension consists of young spontaneously hypertensive rats (SHR). Several studies have showed that early pharmacological treatment in young SHR during the prehypertensive period (4–8 weeks of age) has a prolonged BP-lowering effect and it maintains cardiac protection in adulthood [8,9]. However, young SHR are not a suitable model for representing essential prehypertension in adulthood due to the early onset of fully developed hypertension (i.e., systolic BP over 140 mm Hg) before reaching adulthood (~12 weeks of age) [10]. A more appropriate genetic model of essential prehypertension consists of borderline hypertensive rats (BHR), which were obtained by the mating of female SHR and male normotensive Wistar-Kyoto (WKY), resulting in a systolic BP of approximately 140 mm Hg in the adulthood stage [11,12].

Regarding the mechanisms of (pre)hypertension development, several human studies noticed increased markers of oxidative stress and decreased antioxidant capacity in the plasma of young as well as middle-aged prehypertensive adults [13,14]. Oxidative damage was also observed in adult 22-week-old BHR [11], which suggested that the altered redox state might contribute to the development of (pre)hypertension. Iron, which is an essential nutrient for all cells, in addition to its other functions, contributes to the production of reactive oxygen species (ROS), modulates redox balance [15], and affects nitric oxide bioavailability and, thus, vascular function [16]. However, the role of iron and/or iron-containing compounds in the development of arterial hypertension is not fully understood. For example, studies of Astma et al. [17] and Zhu et al. [18] showed that the haemoglobin levels were positively associated with BP in non-hypertensive people. In addition, a high incidence of systolic BP in prehypertensive range prevailed in young adults, in them haemoglobin level was positively correlating with systolic BP [19]. In contrast, in patients with hereditary haemochromatosis with profound iron overload endothelial function was impaired without changes in BP [20]. Endothelial dysfunction, which results from reduced nitric oxide (NO) bioavailability, is another factor that is involved in the development of (pre)hypertension [21]. In addition, alterations in the renin-angiotensin system and in neurogenic regulation also contribute to the development of (pre)hypertension [22]. 

Several lifestyle-related changes are recommended to reduce elevated BP, such as weight reduction, exercise, and a healthy diet. High attention is paid to the consumption of flavan-3-ols (catechins), as several clinical and epidemiological studies have found that a reduction in the systolic BP is associated with an increased consumption of cocoa products [23], the rich source of Epi. The recent studies have confirmed that Epi is the subject of major in vivo metabolisation and Epi and/or its metabolites are absorbed well from the gastrointestinal tract [24,25]. Furthermore, there are other studies that found Epi and/or its metabolites in plasma in concentrations that are sufficient to produce biological effects [23]. A decrease in the BP, dependent on the Epi content, was found in a meta-analysis that was performed by Ellinger et al. [26]. The BP-lowering effect of Epi was also observed in various experimental studies while using with N(ω)-nitro-L-arginine methyl ester (L-NAME)-induced [27], fructose-induced [28], and deoxycorticosterone acetate (DOCA)–salt-induced hypertension [29]. Epi was also found to induce a reduction in the systolic BP of both young and adult SHR [30,31]. Elevated NOS activity and reduced oxidative stress seem to be the main factors that lead to a BP decrease after long-term Epi treatment. 

However, little is known about the molecular mechanisms that are associated with the elevated NO production and reduced release of ROS during Epi treatments. Nuclear factor erythroid 2-related factor 2 (Nrf2) is a transcription factor that plays a significant role in hypertension development [32,33]. Nrf2 is involved in cellular responses to oxidative stress; it modifies the ROS level by modulating the antioxidant and detoxification outputs as well as it modifies inflammation [33,34,35]. Peroxisome proliferator-activated receptor gamma (PPAR-γ) is transcription factor that modulates several signalling pathways, such as phosphoinositide 3-kinase/protein kinase B/nitric oxide synthase, the renin-angiotensin system, angiotensin-receptor 1/nicotinamide adenine dinucleotide phosphate oxidase pathway, as well as redox homeostasis [36]. Nrf2 and PPAR-γ are both present in the heart and arteries [37,38] and it might be involved in the Epi-induced BP-lowering effect. 

Therefore, this study investigated the effects of (–)-epicatechin on BP, locomotor activity, anxiety-like behaviour, nitric oxide synthase (NOS) activity, and superoxide (O_2_•^–^) production in the aorta, left heart ventricle (LHV), brainstem, and frontal neocortex, as well as on the relative content of iron-containing compounds in the blood of young borderline BHR. In addition, the gene expressions of NO synthase isoforms (*nNOS*, *eNOS*, *iNOS*) and involvement of mechanisms that are mediated by transcription factors Nrf2 and PPAR-γ were investigated in the LHV and aorta. Furthermore, we tested the hypothesis that Epi treatment can result in a long-term reduction of BP, behavioural alterations, and alterations in the abovementioned genes after the cessation of Epi administration.

## 2. Materials and Methods

### 2.1. Animals and Treatment

Five-week-old BHR males, i.e., the offspring of SHR dams and WKY sires, were used in this study. The rats (*n* = 28) were divided into four groups: the seven-week control (C7, *n* = 7), Epi-treated (Epi, *n* = 7), nine-week control (C9, *n* = 7), and post-Epi (PE, *n* = 7) groups. The rats in the Epi and PE groups were treated with Epi (Sigma-Aldrich, Bratislava, Slovakia, Cat. no. E1753) added to drinking water for two weeks. Epi was prepared fresh every day before administration to rats by suspension of Epi in tap water at ~85 °C for 5 min. while using vortex. The calculated volume of this Epi, based on the body weight of rats and drinking volume, was added to the drinking bottles of the rats to reach a final dose of approximately 100 mg/kg/day after drinking all of the liquid during the 24 h period. The rats in both the control groups drank tap water. Rats in the PE group drank tap water for two weeks after the cessation of the Epi administration. The dose of Epi was selected on the basis of our previous study in age-matched SHR, in which the same dose and way of Epi administration elevated antioxidant capacity of plasma and improved aortic NO bioavailability when determined as the elevated NO-dependent component of acetylcholine-induced relaxation [30].

The body weights (BW) of all the rats were measured on the same day as the BP measurements. At the end of the experiment, all of the rats were exposed to brief CO_2_ anaesthesia and subsequently killed by decapitation. After decapitation, the relative weight of the left heart ventricle (LHV/BW) was calculated. Increasing the LHV/BW ratio served as a marker of left-heart hypertrophy. We also measured the relative weight of the left and right kidneys (LK + RK/BW). The Department of Animal Wellness, State Veterinary and Food Administration of the Slovak Republic approved all of the procedures in accordance with the EU Directive 2010/63/EU, decision No. Ro-2561/12-221. 

### 2.2. Open-Field Test

Locomotor activity and anxiety-like behaviour were measured while using the open-field test (OF) between 07:30 a.m. and 10:00 a.m. with a video tracking system called Any-maze (Stoelting, Ireland). At the beginning of the experiment, all of the rats were tested at the age of five weeks (Basal, *n* = 28). After the basal behavioural testing, the rats were assigned to the control (C7, C9) or Epi-treated groups (Epi, PE). The behavioural tests were conducted on day 13 for the C7 and Epi groups and day 27 for the C9 and PE groups. Kluknavsky et al. have previously described the testing conditions in detail [30]. The total distance travelled and total time of immobility in the OF were determined as the parameters of locomotor activity. As markers of anxiety-like behaviour, total distance and relative distance travelled in the central zone (calculated as the percentage of central zone distance with respect to the total distance travelled) were determined.

### 2.3. Systolic Blood Pressure 

The systolic BP was measured in preconditioned, conscious rats while using non-invasive tail-cuff plethysmography between 08:00 a.m. and 11:00 a.m., as previously described by Puzserova et al. [39]. Each value was calculated as the average of five measurements. The BP values were measured at the beginning of the experiment (Basal), and then on the days 3, 7, 14, 17, 21, and 28 of the experiment.

### 2.4. Determination of the Relative Iron Content in Blood

A Quantum Design MPMS-XL 7AC (SQUID) magnetometer with a reciprocating sample operation option (with differential sensitivity of 10^−11^ Am^2^ up to 0.25 T and 10^−10^ Am^2^ up to 7 T) was used to compare the relative content of iron and iron-containing compounds in the blood samples. The advantage of this method is its ability to determine all of the iron forms in a small sample of biological material, with high sensitivity [40,41,42]. 

After decapitation, trunk blood was collected in Eppendorf test tubes and then stored at −80 °C. Subsequently, the blood was defrosted and homogenised using an ultrasonic bath for 60 s (50 kHz, 30 W). After homogenisation 10 μL of the blood was placed on a 16 cm × 6 mm strip of standard white office paper (80 g/m^2^), vacuum-dried, and inserted into a plastic measuring tube [42]. Thus, we obtained a dry sample of the blood in a homogeneous ambient without the need for a gelatine capsule, which is usually the source of an additional magnetic signal. The magnetic characteristics of the samples were measured in the form of isothermal hysteresis curves [40] at a temperature of 2 K (−271.15 °C) and at a magnetic field up to 7 T when the saturation magnetisation (*M_s_*) was reached. *M_s_* is the parameter for determining the concentration of magnetic particles in the biological samples, of which iron is a dominant component under the conditions used in this study. Changes in the magnetic forms of iron, which result from the various sizes of the iron-containing compounds, can be characterised by alterations in the remanent magnetisation (*M_r_*) and coercivity (*H_c_*) [43]. The magnetic properties of the blood were determined for the C7 and Epi groups (*n* = 5 per group).

### 2.5. Superoxide Production

The production of superoxide was measured in the LHV and thoracic aorta (15–20 mg) samples with lucigenin (50 μmol/L)-enhanced chemiluminescence while using a TriCarb 2910TR liquid scintillation analyser (Perkin Elmer, Waltham, MA, USA), as described previously by Slezak et al. [44]. The results are expressed in the form of cpm/mg of tissue.

### 2.6. Nitric Oxide Synthase Activity

The total NOS activity was measured in the 20% tissue homogenates of the LHV, aorta, brainstem, and frontal neocortex by determining the [^3^H]-L-citrulline formation from [^3^H]-L-arginine (ARC, St. Louis, MO, USA), as described previously [39], and expressed in terms of pmol/min./mg of tissue protein. The protein concentration was determined while using the Lowry method.

### 2.7. Gene Expression

The expression levels of *Nrf2*, *PPAR-γ*, neuronal NOS (*nNOS*, isoform I), inducible NOS (*iNOS*, isoform II), endothelial NOS (*eNOS*, isoform III), and *ß-actin* (housekeeping gene) were determined while using a real-time quantitative polymerase chain reaction (RT-qPCR). The total RNA of the thoracic aorta, LHV, brainstem and frontal neocortex were isolated using the TRIsure reagent (Bioline, United Kingdom), according to the manufacturer’s protocols. The amount of total isolated RNA was spectrophotometrically quantified at 260/280 nm while using a NanoDrop spectrophotometer (Thermo Scientific, Waltham, MA, USA). Reverse transcription was performed using 1 μg of the total RNA of each sample and 20 μL of the reaction medium using a SensiFAST™ cDNA Synthesis Kit (Bioline, London, United Kingdom), in accordance with the manufacturer’s protocols (Eppendorf Mastercycler, Hamburg, Germany). SensiFAST mix (SensiFAST SYBR No-ROX kit, Bioline, London, UK) was used for gene amplification. The preparation of the PCR mixture, thermal cycling conditions, used apparatus, and detection software were described previously [30]. Gene-specific primers were designed while using the PubMed database (Gene) and program (Primer-BLAST). The primer pairs used to amplify the nNOS (GenBank accession No. NM_052799.1), iNOS (GenBank accession No. NM_012611.3), eNOS (GenBank accession No. NM_021838.2), PPAR-γ (GenBank accession No. NM_013124.3) and Nrf2 (GenBank accession No. NM_031789.2) genes, as well as β-actin (GenBank accession No. NM_031144.3, a housekeeping gene) are listed in Table 1. The samples were measured using the Bio-Rad CFX Manager software 2.0 (Hercules, CA, USA). The gene expressions were considered as the ratio of the given gene’s expression to β-actin expression.

All of the chemicals used in this study were purchased from Sigma-Aldrich (Bratislava, Slovakia) and Merck Chemicals (Bratislava, Slovakia), unless stated otherwise.

### 2.8. Statistical Analysis

The BP results were separately analysed for the Epi-treatment period (*n* = 14 in each group) and post-treatment period (*n* = 7 in each group) while using a two-way ANOVA (treatment x day of experiment). The saturation magnetisation of the blood was analysed using the Student’s t-test. All of the other data were analysed with a one-way ANOVA. All of the ANOVA analyses were followed by Bonferroni post-hoc tests. The correlations between the gene expressions were determined using Pearson’s correlation coefficient (*r*). The values were found to significantly differ when *p* < 0.05. The data were presented in the form of mean ± standard error of the mean (SEM). GraphPad Prism 5.0 (GraphPad Software, Inc., San Diego, CA, USA) was used for the statistical analyses.

## 3. Results

There were age-dependent changes in the BW and relative kidney mass (LK + RK/BW), and Epi had no effect on these parameters when compared to the age-matched controls (Table 2). No alterations were found in the relative LHV mass (LHV/BW) among the groups (Table 2). The saturation magnetisation of the Epi-treated rats was significantly reduced by approximately 54% vs. the age-matched control group. No significant changes were found in the remanent magnetisation and coercivity (Table 2).

**Figure 1:**
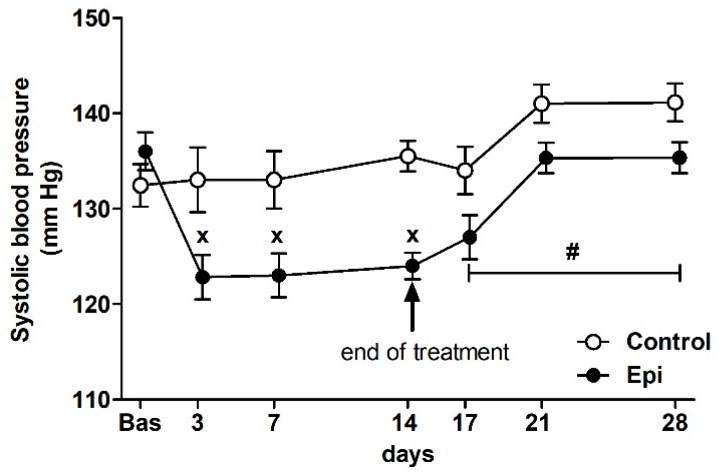
The effect of (–)-epicatechin on the systolic blood pressure of borderline hypertensive rats. The values represent the mean ± SEM. ^x^ *p* < 0.05 vs. control group on the respective day of experiment, ^#^ *p* < 0.03 vs. control group (ANOVA main effect of group during post-treatment period i.e., on days 17, 21 and 28, see Results). Abbreviations. Bas: baseline value, Epi: (–)-epicatechin group.

There were no significant differences in the basal BP of rats that were assigned to the control groups (132 ± 2 mm Hg, *n* = 14) and Epi-treated groups (137 ± 2 mm Hg, *n* = 14) at the beginning of the study (Figure 1). The ANOVA analysis confirmed significant differences in BP between the groups during the treatment (F_1,100_ = 19.03, *p* < 0.0001, main effect of treatment), the significant effect of the day of treatment (F_3,100_ = 4.08, *p* < 0.01, main effect of day), as well as the significant effect of the interaction of treatment and day of experiment (F_3,100_ = 5.80, *p* < 0.002) during the treatment period. Epi significantly reduced the BP during the treatment when compared to the control group. During the post-treatment period (i.e., days 17, 21, and 28), the ANOVA showed significant difference in BP between the control and PE groups (F_1,35_ = 10.65, *p* < 0.003, main effect of post-treatment) and the significant effect of the day of experiment (F_2,35_ = 6.95, *p* < 0.003, main effect of day). 

**Figure 2:**
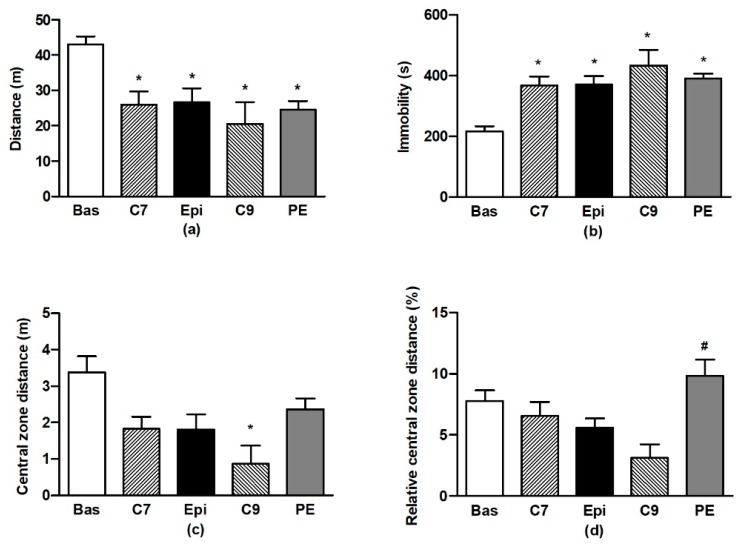
The effect of (–)-epicatechin on the behaviours of borderline hypertensive rats. Total distance travelled (**a**) and total immobility (**b**) in the open field; total distance (**c**) and relative distance (**d**) travelled in the central zone of the open field. The values represent the mean ± SEM. * *p* < 0.05 vs. the basal group value, ^#^ *p* < 0.05 vs. the C9 group. Abbreviations. C7: seven-week-old control group, C9: nine-week-old control group, Epi: (–)-epicatechin group, PE: (–)-epicatechin post-treatment group.

The ANOVA analysis confirmed significant differences in OF behaviour during testing for all of the distance travelled (F_4,59_ = 7.76, *p* < 0.001), immobility (F_4,59_ = 12.5, *p* < 0.001), distance travelled in the central zone (F_4,59_ = 3.83, *p* < 0.008), and relative distance travelled in the central zone (F_4,59_ = 2.9, *p* < 0.03). Repeated testing of BHR in OF led to the habituation of locomotor activity and immobility (Figure 2a,b) when compared to the respective basal values. There were no differences in the individual values determined for the Epi group as compared to the age-matched controls (C7). However, for the PE group, a lack of habituation (vs. the basal value) was found in distance travelled in the central zone, in contrast to the age-matched C9 control group (Figure 2c). This resulted in the significant increase in relative distance travelled in the central zone for the PE group as compared to the age-matched (C9) control group (Figure 2d).

**Figure 3:**
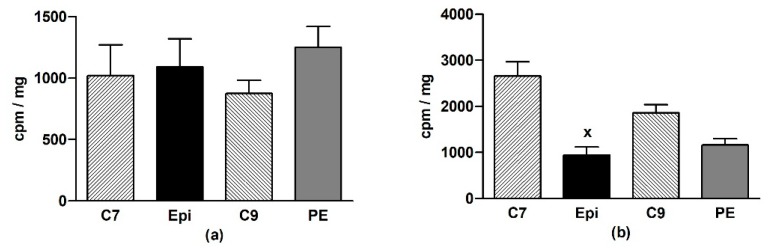
The effect of (–)-epicatechin on the superoxide production in the left heart ventricle (**a**) and aorta (**b**) of borderline hypertensive rats. The values represent the mean ± SEM. ^x^ *p* < 0.001 vs. C7 group, *n* = 6–7 per group. Abbreviations. C7: seven-week-old control group, C9: nine-week-old control group, Epi: (–)-epicatechin group, PE: (–)-epicatechin post-treatment group.

In the LHV, Epi did not change O_2_•^–^ production as compared to the age-matched control groups (Figure 3a). In the aorta, a significant (*p* < 0.001) reduction of O_2_•^–^ production was detected in the Epi group when compared to the C7 group, and a similar tendency was seen in the post-treatment period (Figure 3b).

**Figure 4:**
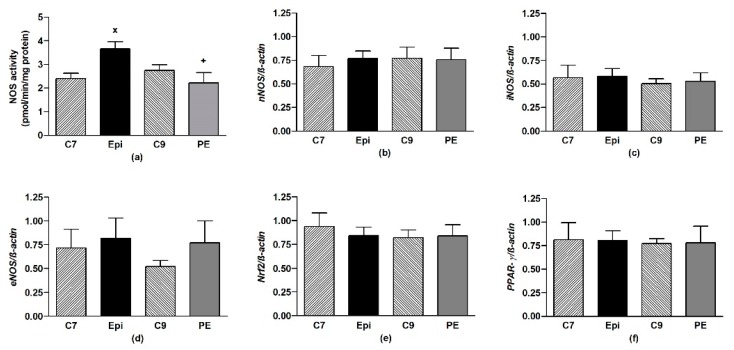
The effect of (–)-epicatechin on the nitric oxide synthase (NOS) activity (**a**) and gene expression of neuronal *nNOS* (**b**), inducible *iNOS* (**c**), endothelial *eNOS* (**d**), Nrf2 (**e**) and *PPAR-γ* (**f**) in the left heart ventricle of borderline hypertensive rats. The values represent the mean ± SEM, *n* = 6–7 per group. ^x^ *p* < 0.05 vs. C7 group, ^+^ *p* < 0.05 vs. Epi group. Abbreviations. C7: seven-week-old control group, C9: nine-week-old control group, Epi: (–)-epicatechin group, PE: (–)-epicatechin post-treatment group, *Nrf2*: nuclear factor erythroid 2-related factor 2, *PPAR-γ*: peroxisome proliferator-activated receptor gamma.

The Epi-treatment significantly (*p* < 0.05) increased the total NOS activity in the LHV, returning back to the control levels after cessation of the treatment (Figure 4a). Epi failed to alter the gene expressions of *eNOS*, *nNOS*, *iNOS*, as well as *Nrf2* and *PPAR-γ* in the LHV (Figure 4b–f). 

**Figure 5:**
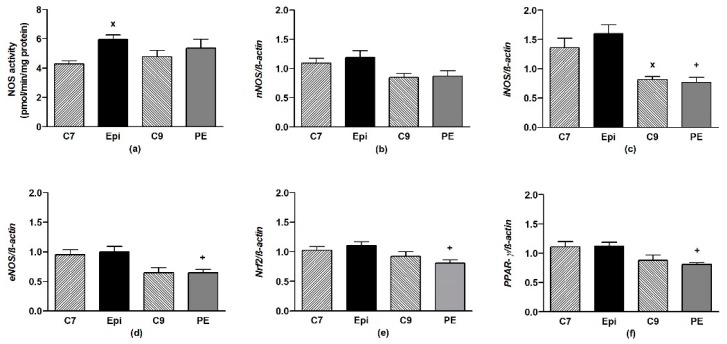
The effect of (–)-epicatechin on the nitric oxide synthase (NOS) activity (**a**) and gene expression of neuronal nNOS (**b**), inducible iNOS (**c**), endothelial eNOS (**d**), Nrf2 (**e**) and PPAR-γ (**f**) in the aorta of borderline hypertensive rats. The values represent the mean ± SEM; n = 6–7 per group. ^x^ *p* < 0.05 vs. C7 group, ^+^ *p* < 0.05 vs. Epi group. Abbreviations: C7: seven-week-old control group, C9: nine-week-old control group, Epi: (–)-epicatechin group, PE: (–)-epicatechin post-treatment group, Nrf2: nuclear factor erythroid 2-related factor 2, PPAR-γ: peroxisome proliferator-activated receptor gamma.

Epi significantly (*p* < 0.04) increased the total NOS activity in the aorta as compared to C7 (Figure 5a), despite the gene expressions of the individual NOS isoforms remaining unchanged (Figure 5b–d). During the post-treatment period, the NOS activity and gene expressions were similar to those in the age-matched C9 control group.

There were significant correlations between the gene expressions of *Nrf2* and *PPAR-γ*, *eNOS*, *iNOS*, and *nNOS*, as shown in Table 3.

**Figure 6:**
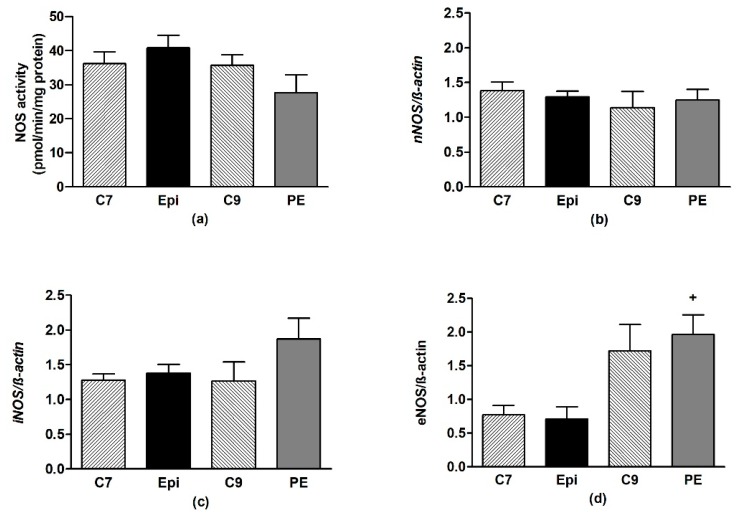
The effect of (–)-epicatechin on the nitric oxide synthase (NOS) activity (**a**) and gene expression of neuronal nNOS (**b**), inducible iNOS (**c**) and endothelial eNOS (**d**) in the frontal neocortex of borderline hypertensive rats. The values represent the mean ± SEM. ^+^ *p* < 0.05 vs. Epi group. Abbreviations. C7: seven-week-old control group, C9: nine-week-old control group, Epi: (–)-epicatechin treatment group, PE: (–)-epicatechin post-treatment group.

**Figure 7:**
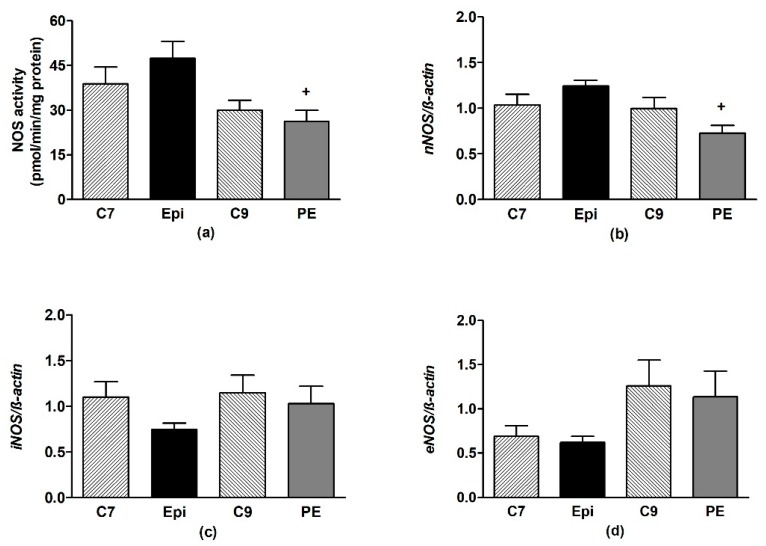
The effect of (–)-epicatechin on the nitric oxide synthase (NOS) activity (**a**) and gene expression of neuronal *nNOS* (**b**), inducible *iNOS* (**c**) and endothelial *eNOS* (**d**) in the brainstem of borderline hypertensive rats. The values represent the mean ± SEM. ^+^ *p* < 0.05 vs. Epi group. *Abbreviations*. C7: seven-week-old control group, C9: nine-week-old control group, Epi: (–)-epicatechin group, PE: (–)-epicatechin post-treatment group.

In the frontal neocortex and brainstem, Epi had no effect on the NOS activity or expression of the individual NOS isoforms as compared to the respective age-matched control group (Figure 6 and Figure 7). 

The Pearson’s correlation coefficients (*r*) for the gene expressions of the nuclear factor erythroid 2-related factor 2 (*Nrf2*), endothelial (*eNOS*), neuronal (*nNOS*), inducible (*iNOS*), nitric oxide synthase and peroxisome proliferator-activated receptor-γ (*PPAR-γ*), respectively.

## 4. Discussion

The effects of an increased intake of cocoa products or Epi and Epi-containing foods are under investigation, not only in the area of BP regulation, but also in relation to the various neuropsychological and mood disorders in humans [23]. There are currently no studies investigating the modulation of locomotor activity and anxiety-like behaviour resulting from purified Epi or Epi-containing foods in BHR rats. BHR, which is an experimental model of human essential prehypertension, might inherit a predisposition to locomotor hyperactivity from the SHR parent [45]. However, the results of this study showed that Epi failed to significantly affect the OF behaviour of young BHR after two-week treatment in contrast to the reduced locomotor activity and relative distance travelled in the central zone previously found in the age-matched Epi-treated SHR [30]. However, the low habituation level for distance travelled in the central zone and increased relative distance travelled in the central zone at two weeks post treatment suggest a delayed anxiolytic effect of Epi in young BHR. In a study with normotensive Wistar rats, a single-dose of cocoa had an anxiolytic effect with unchanged locomotor activity, whereas a two-week treatment period failed to affect the behaviour of the rats [46]. In a study with Wistar-Unilever rats, the administration of cocoa extract for two weeks had antidepressant effects [47]. The results currently available in the literature suggest that Epi has ambiguous effects on behaviour, which probably depends on the animal model used. However, the literature has also indicated the penetration of Epi and its metabolites into the brain [48]. Further studies are required for clarifying the mechanisms of Epi’s action on the spontaneous behaviour of rats and its possible association with blood pressure.

On the other hand, the BP-lowering effects of Epi are well known. In this study, the five-week BHR had a systolic BP of ~134 mm Hg, which slowly increased to ~141 mm Hg through the course of this experiment (in untreated rats). These values are similar to those that were observed in previous studies in adult BHR [11,12,49]. Regarding the development of BP in young BHR males, our results suggest the absence of a critical BP developmental window associated with the rising BP at the age of 5–7 weeks, in contrast to that in young SHR [50]. 

The depressor effect of Epi was observed during the entire period of treatment, followed by a gradual increase in the BP after cessation of treatment. This study is, to our knowledge, the first one that aimed at investigating the long-term effects of Epi in BHR after the cessation of the Epi treatment. The long-term influence of Epi on BP was studied on the basis of previous studies that showed long-term beneficial effects of antihypertension treatment in young prehypertensive SHR [8,9]. We found that the lower values of BP persisted two weeks after the cessation of the Epi treatment (statistically determined as the main effect), which might have been a result of the prevention of BP-induced vascular and/or organ changes that cause postponed BP increase. 

Several human studies have examined the effects of flavanol-rich cocoa products associated with high content of Epi in prehypertensive individuals, despite the lack of animal studies. Petyaev et al. described the depressoric effects of cocoa products in prehypertensive subjects after two weeks of consuming 30 g dark chocolate that contained 85% cocoa [51]. Similar findings were reported for prehypertensive subjects after 15 days of consuming 30 g dark chocolate that contained 70% cocoa [52]. On the other hand, Ried et al. observed unaltered systolic BP in prehypertensive subjects after eight weeks of consuming 50 g dark chocolate that contained 70% cocoa [53]. The variability of the results that were obtained from those studies can be associated to the differing ages of the subjects, lengths of the treatment and, particularly, the variable flavanol content used in the individual studies. 

Oxidative stress, which iron plays an important role in causing, can also result in the development of elevated BP [34,54,55]. In our study, Epi significantly reduced the level of iron-containing compounds in blood; reduced saturation magnetisation was observed while using SQUID magnetometry. However, the size of the iron particles and/or their chemical moiety seems to be unchanged, as coercivity and remanent magnetisation remained unaltered. SQUID magnetometry is a novel approach to quantify different iron forms in biological samples with high sensitivity that might provide new information for the understanding of the pathomechanisms of various diseased states as described previously [40,43]. The relative reduction of iron-containing compounds in Epi-treated vs. control rats in this study might be related to the inhibition of iron absorption in the enterocytes by Epi or its metabolites [56]. Indeed, there are animal and human studies that found reductions in iron bioavailability after the consumption of tea or various polyphenols-rich foods [57]. In our study, we showed the reduced levels of iron and/or iron-containing compounds in the blood of Epi-treated BHR. However, the exact mechanisms and chemical moiety of iron-containing compounds that are affected by Epi remain to be elucidated.

The lowered iron content level might be also involved in the reduced vascular ROS production. In our study, Epi significantly reduced the O_2_•^–^ production in the aorta. This antioxidant effect was seen to partially persist after the cessation of the treatment, as the O_2_•^–^ production in the aorta of the PE group was similar to that of the Epi group. The Epi-reduced O_2_•^–^ production in the aorta of BHR was similar to our previous findings in the young SHR [39]. The antioxidant effects of Epi may also be a result of the direct scavenging of ROS [58], activation of antioxidant enzymes [59], or inhibition of pro-oxidant enzymes [58,60]. The suppression of the oxidative damage caused by Epi was also observed in the SHR [30] and other experimental models of hypertension [27,28,29]. 

It is well known that NO plays a key role in the modulation of BP [21]. In this study, the Epi stimulated NO production in the aorta and LHV (in contrast to the brain regions) of the young BHR, which, however, did not persist post treatment. The increased NO production due to Epi was found in various experimental models of hypertension: young SHR [30], adult SHR [31], as well as L-NAME-induced and fructose-induced hypertension [27,28] models. The observed NOS-stimulating effect of Epi is also supported by human studies in which the long-term administration of dark chocolate led to an increase of NO metabolites in blood in prehypertensive and hypertensive probands [52,61].

An important aim of our study was to investigate the genomic effects of the Epi during the stimulation of NO release and antioxidant defence in the LHV and aorta. The mRNA of all eNOS, iNOS and nNOS was present in the LHV and aorta in BHR. There are studies that found a significant role of nNOS-derived NO in local physiologic regulation of vascular tone [62], as well as in the physiological regulation of basal systemic vascular resistance and BP in healthy humans [63], despite the well-known role of eNOS-produced NO in flow-mediated dilatation. We found that the Epi did not alter the gene expression of the individual NOS isoforms and transcription factor *Nrf2* in the LHV and aorta. This suggests that the antioxidant effects of Epi in the abovementioned tissues are not related to the improved gene expressions of Nrf2-activated antioxidant enzymes. In addition, Nrf2 is involved in the NO-mediated regulation of iron metabolism, as it upregulates ferritin and ferroportin transcriptions, which results in the reduction of intracellular iron content [64]. However, these mechanisms seem to be less plausible in the cardiovascular system, as the *Nrf2* gene expression was unaltered during this experiment. Furthermore, Epi does not seem to modify PPAR-γ-mediated lipid metabolism and energy balance in the LVH and aorta [65] as *PPAR-γ* gene expressions were also found unaltered. There were significant correlations between *Nrf2* and *PPAR-γ*, as well as the individual NOS isoforms, despite the unchanged gene transcriptions, which suggests the regulatory role of Nrf2 in NOS expressions in the heart and aorta; this mechanism remained unaltered by Epi. The coordinating role of *Nrf2* in *PPAR-γ* expressions and endothelial NO generation was previously found by Luo et al. [66]. Regarding Epi effects, relatively few in vivo and in vitro studies have investigated the genomic and proteomic effects of purified Epi, especially the expressions of the genes that were investigated in this study. Our previous study showed that the expressions of all the NOS isoforms in the LHV of young SHR were unchanged [30], which was similar to those that were found in the young BHR. Mohamed et al. showed that *iNOS* gene expression was unaltered by Epi treatment in the brain of Wistar rats [67]. A study by Gómez-Guzmán et al. showed increased gene and protein expressions of Nrf2 upon Epi administration in the aorta of Wistar rats, which is in contrast to our findings [29]. Another study found the eNOS protein expression to be unchanged in Sprague-Dawley rats simultaneously treated with L-NAME and Epi [68]. Prince et al. investigated the alteration of the eNOS protein expression by administering Epi in the kidneys of Sprague-Dawley rats [69]. Rats that were simultaneously fed with fructose and Epi had similar expression levels as those of fructose-fed rats, which indicated that Epi does not affect the eNOS expression in the kidney [68]. Furthermore, Gómez-Guzmán et al. found the eNOS protein expression to be unaltered by Epi in the aorta of Wistar rats [70]. Thus, the BP-lowering effect of Epi was probably related to the increase of NOS activity and/or NO bioavailability due to improved redox conditions in the cardiovascular system of young BHR, similarly as it was found in SHR [30], without genomic effects being involved in the stimulation of NO release or antioxidant defence.

## 5. Conclusions

Our study showed that a two-week oral treatment of young BHR with Epi had a BP-lowering effect that persisted for two weeks after the cessation of the treatment. This was associated with a delayed reduction of anxiety-like behaviour. The mechanism underlying the BP-lowering effect of Epi was associated with the reduced relative content of the iron-containing compounds in the blood, reduced O_2_•^–^ production, and stimulation of NOS activity in the LHV and aorta without increasing the mRNA expressions of the individual NOS isoforms or *Nrf2* and *PPAR-γ* transcription factors. In addition, our results suggest that continuous Epi treatment is required for maintaining elevated NO synthase activity and redox balance in the heart and aorta of young BHR. However, caution is needed due to the possible reduction of blood iron content with a long-term Epi intake.

## Figures and Tables

**Table 1:** Primer pairs used to amplify selected genes.

Gene	Forward (Sense) Primer	Reverse (Antisense) Primer	Temp
*eNOS*	CGGCGCAAAAGGAAGGAATC	CCAGCCCAAACACACAGAAC	60 °C
*iNOS*	TGGAGGTGCTGG AAGAGTT	GGAGGAGCTGATGGAGTAGT	57 °C
*nNOS*	CGCTACGCGGGCTACAAGCA	GCACGTCGAAGCGGCCTCTT	60 °C
*Nrf2*	AGGTTGCCCACATTCCCAAA	TATCCAGGGCAAGCGACTCA	60 °C
*PPAR-γ*	TCCCGTTCACAAGAGCTGAC	GCTCTACTTTGATCGCACTTTGG	60 °C
*β-actin*	AATCGTGCGTGACATCAAAG	ATGCCACAGGATTCCATACC	57 °C

**Table 2:** Basic biometric parameters and magnetic properties of blood.

Group	BW (g) *n* = 7	LHV/BW (mg/g) *n* = 7	(LK + RK)/BW (mg/g) *n* = 7	*M_s_* (10^−3^ Am^2^/kg) *n* = 5	*M_r_* (10^−2^ Am^2^/kg) *n* = 5	*H_c_* (A/m) *n* = 5
C7	178 ± 5.9	1.98 ± 0.10	9.33 ± 0.18	210 ± 13.85	0.19 ± 0.07	1569 ± 727
Epi	177 ± 2.1	1.89 ± 0.09	9.40 ± 0.26	98 ± 26.26 ^x^	0.08 ± 0.01	1260 ± 244
C9	249 ± 4.6 ^x^	1.84 ± 0.04	8.07 ± 0.09 ^x^	N.D.	N.D.	N.D.
PE	244 ± 4.0 ^+^	1.95 ± 0.05	8.11 ± 0.05 ^+^	N.D.	N.D.	N.D.

Abbreviations: C7: seven-week-old control group, C9: nine-week-old control group, Epi: (–)-epicatechin group, PE: (–)-epicatechin post-treatment group, BW: body weight, LHV: left heart ventricle, LK: left kidney, RK: right kidney, *M_s_*: saturation magnetisation of the blood samples, *M_r_*: remanent magnetisation of blood samples, *H_c_*: coercivity of blood samples, N.D.: not determined. The values represent the mean ± SEM. ^x^ *p* < 0.001 vs. C7, ^+^ *p* < 0.001 Epi.

**Table 3:** Correlations between the gene expressions in the aorta and left heart ventricle.

	*Nrf2*					
	Aorta			Left Heart Ventricle		
	*r*	*p* <	*n*	*r*	*p* <	*n*
*PPAR-γ*	0.896	0.0001	26	0.714	0.0001	24
*eNOS*	0.772	0.0001	28	0.719	0.0001	24
*nNOS*	0.694	0.0001	27	0.570	0.004	24
*iNOS*	0.689	0.0001	27	0.508	0.01	25
